# The Intricate Process of Calcification in Granuloma Formation and the Complications Following *M. tuberculosis* Infection

**DOI:** 10.3390/biom15071036

**Published:** 2025-07-17

**Authors:** Nickolas Yedgarian, Jacqueline Agopian, Brandon Flaig, Fouad Hajjar, Arshavir Karapetyan, Kannan Murthy, Ani Patrikyan, Kirakos Tomas, Kevin Tumanyan, Mohammad J. Nasiri, Selvakumar Subbian, Vishwanath Venketaraman

**Affiliations:** 1 College of Osteopathic Medicine of the Pacific, Western University of Health Sciences, Pomona, CA 91766, USA; nickolas.yedgarian@westernu.edu (N.Y.); jacqueline.agopian@westernu.edu (J.A.); brandon.flaig@westernu.edu (B.F.); fouad.hajjar@westernu.edu (F.H.); arshavir.karapetyan@westernu.edu (A.K.); kannan.murthy@westernu.edu (K.M.); ani.patrikyan@westernu.edu (A.P.); 2 College of Podiatric Medicine, Western University of Health Sciences, Pomona, CA 91766, USA; kirakos.tomas@westernu.edu (K.T.); kevin.tumanyan@westernu.edu (K.T.); 3 Department of Microbiology, School of Medicine, Shahid Beheshti University of Medical Sciences, Tehran 19839-69411, Iran; mj.nasiri@hotmail.com; 4 Public Health Research Institute, New Jersey Medical School, Rutgers University, Newark, NJ 07103, USA; subbiase@njms.rutgers.edu

**Keywords:** *Mycobacterium tuberculosis*, calcification, fibrosis, granuloma

## Abstract

*Mycobacterium tuberculosis*—an acid-fast staining bacterium—is a serious global health challenge that can have both short-term and long-term complications. Although the immune response helps trap the infection, it can also cause necrosis and calcification, leading to lung tissue damage. Calcification is a known outcome of chronic granuloma evolution in TB. Multiple pathways contribute to fibrosis and calcification; some examples are IL-1β, TGF-β, and TNF-α. Current antifibrotic drugs, such as nintedanib and pirfenidone, are effective but may increase the risk of latent tuberculosis reactivation in certain patients. Experimental therapies such as artemisinin derivatives have shown promise in preclinical TB fibrosis models, while cell-based therapies like bone marrow-derived mononuclear cells are also under early investigation for dual antifibrotic and immunomodulatory effects. This literature review will explore recent studies on the pathogenesis of *M. tuberculosis*, the mechanisms underlying calcification in granuloma formation, and subsequent complications of the disease process.

## 1. Introduction

Tuberculosis (TB) is a significant global health challenge, with 1.3 million deaths annually (World Health Organization [WHO], 2023). *M. tuberculosis* is a Gram-positive, acid-fast bacillus transmitted via aerosol droplets. Its spread is easily facilitated as it is usually suspended in air for prolonged periods of time [1].

Understanding the early events following infection with *M. tuberculosis* is critical to elucidating how TB progresses and how the immune system works to contain the infection. Initially, upon inhalation of aerosolized *M. tuberculosis* particles, the bacteria reach the terminal bronchioles and alveoli, where alveolar epithelial cells recognize the pathogen and present it to macrophages. Pulmonary M cells have been identified as the chief facilitators of *M. tuberculosis* transcytosis across the alveolar epithelium. This transports the bacteria to regional lymph nodes, which initiates an adaptive immune response [2]. In most individuals that are immunocompetent, this leads to granuloma formation—organized cellular structures formed by the immune system that restrict bacterial replication. This containment represents latent TB, where *M. tuberculosis* persists in a dormant state without actively replicating and causing disease. Failure to control bacterial replication can lead to primary TB, characterized by uncontrolled bacterial growth and pulmonary tissue damage.

Critical to disease containment is the host immune response, particularly CD4+ T helper cells (Th1) orchestrating granuloma formation. CD4+ T cells are activated upon antigen presentation via major histocompatibility complex class II (MHC II) molecules. This is facilitated by the interaction between the T cell receptor and MHC II, along with other co-stimulatory signals [3]. Key cytokines such as interferon-gamma (IFN-γ), interleukin-12 (IL-12), and tumor necrosis factor-alpha (TNF-α) play a central role in granuloma formation and maintenance. IL-12 promotes Th1 differentiation, IFN-γ activates macrophages to enhance antimicrobial activity, and TNF-α supports immune cell recruitment and granuloma stability [4]. These immune mechanisms collectively contribute to *M. tuberculosis* containment within granulomas. But the duality of this immune response that aims to contain the infection is what leads to lung pathology.

While granulomas serve to limit infection, they can also contribute to long-term lung damage—even in individuals who clear the infection. The impact of *M. tuberculosis* infection extends beyond just active and latent disease. Even after successful treatment of the disease, a significant portion of TB survivors suffer from post-TB lung disease (PTLD), which is characterized by persistent respiratory impairments. Some examples include fibrosis, bronchiectasis, and chronic airflow obstruction [4]. The underlying pathophysiology of PTLD involves long-term structural lung damage resulting from the host immune response, inflammation, and delayed bacterial clearance. Understanding these complications is essential in addressing the complete aspects of TB-related morbidity.

Given the complex interplay between host immunity and bacterial persistence, this review will examine the immune mechanisms driving *M. tuberculosis* infection and explore the role of calcifications in granuloma formation relating to bacterial containment.

## 2. Materials and Methods

Data were collected on the calcification and complications of *M. tuberculosis* infections using PubMed databases and Zotero version 6.0.37. Zotero is a bibliography management software program. Key words included “*Mycobacterium tuberculosis*”, “*M. tuberculosis*, calcification”, “*M. tuberculosis*, fibrosis”, “*M. tuberculosis*, granuloma”, “*M. tuberculosis*, *mechanism”,* “TB, complications” “TB treatments”, and “TB, imaging”. We included studies published up to 2025. We included peer-reviewed English-language studies involving humans or animals that addressed tuberculosis pathogenesis, calcification, fibrosis, imaging, or treatment. We excluded non-English publications, editorials, letters, conference abstracts, and studies not directly relevant to tuberculosis complications or fibrosis.

## 3. Mechanisms of Calcification in Granuloma Formation

When infected with *M. tuberculosis*, there are variable immunological methods that the human body uses to interact with the infection. In immunocompromised people, *M. tuberculosis* can cause symptomatic primary infection. However, in the majority of cases, people are asymptomatic and have latent tuberculosis granulomas that are waiting for the immune response to diminish in order for the granuloma to liquify and for tuberculosis to be reactivated [5].

**Figure 1:**
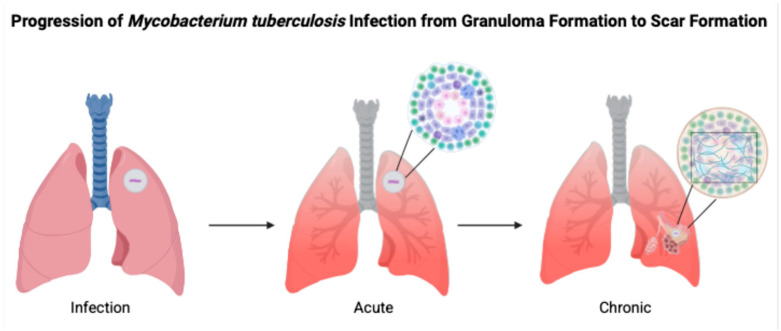
(Created in BioRender). Progression of Mycobacterium tuberculosis infection from granuloma formation to scar formation. Infection: Initial infection with Mycobacterium tuberculosis. Acute: CD4+ T helper cells initiate granuloma formation by stimulating TNF-α. Chronic: Chronic inflammation and reactive oxygen species (ROS) release nitric oxide and free radicals, stimulating IL-12 and IFN-γ, leading to bacterial dormancy, collagen formation, and fibrosis. Prolonged Chronic: Prolonged inflammation results in calcified scar formation and fibrosis, encapsulating granuloma. Figure 1 was created with Biorender.com (accessed on 12 April 2025).

Granuloma formation is a part of the human body’s immunological response to the primary infection. Lung tissue cells and leukocytes produce chemokines and cytokines which are responsible for managing granuloma formation [6]. With the containment of *M. tuberculosis* in the granuloma, the surrounding tissue often undergoes caseous necrosis and becomes enriched with calcium salts. This process is facilitated by activated macrophages, particularly under the influence of Th1 cytokines like interferon-gamma (IFN-γ) which enhance intracellular bactericidal activity through nitric oxide production and phagolysosome maturation. These immune responses help wall off the pathogen, allowing it to persist in a dormant state—a condition known as latent tuberculosis [5]. As can be seen in Figure 1, infection with *M. tuberculosis* moves through acute to chronic stages through various mechanisms involving TNF-α, IL-12, and IFN-γ.

## 4. Granulomas That Cause Calcification and Granulomas That Do Not

Infection with *M. tuberculosis* results in caseating granulomas in lymph nodes. In contrast, in sarcoidosis, necrosis and caseation would not be expected [7]. This is a key distinction used in differential diagnoses. A study from 1996 by Moon et al. describes four progressive stages of active TB lymphadenitis. Stage 1 aligns with lymphoid hyperplasia with granulomas but without caseation necrosis, while stages 2 and 3 involve caseation necrosis [8]. Stage 4 is rare with mediastinal disease and involves rupture of the caseating component. Nodal types are classified into three categories based on homogeneity and what is seen on MRI both with and without contrast. Type 1 is homogeneous with hyperintensity and patients have mild signs and symptoms. Type 2 is not homogeneous with hyperintensity and patients experience traditional signs of TB, while Type 3 nodes are homogenous and hypointense, corresponding to fibrocalcification, with patients generally being asymptomatic [8]. According to that study, stage 1 corresponds with Type 1 nodes, stage 2 and 3 with Type 2 nodes, and type 3 nodes with fibrocalcification [8]. This correlation between node type, stage of disease, and patient presentation is important for clinical management of tuberculous lymphadenitis.

In fibrocalcific nodes, different calcification patterns can be seen. Typically, the pattern of calcification can give insight into the progression or processes involved in the disease. In a study by Gawne-Cain and Hansell, a comparison between calcification patterns was made between two granulomatous diseases: TB and sarcoidosis. The proposed cause of the differences in calcification patterns is likely due to the caseation and necrosis involved in tuberculosis that does not occur with sarcoidosis [7]. In this study, 46% of patients diagnosed with TB showed calcification in their lymph nodes and 40% of patients who were diagnosed one year prior to imaging showed calcifications. Common calcification patterns seen in granulomas within lymph nodes include diffuse/homogenous (group I), focal (group II), eggshell (group III), and complete calcifications (group IV). Complete calcifications were more common in patients diagnosed with tuberculosis than in those with sarcoidosis, while focal calcifications were more common in sarcoidosis patients than in TB patients (*p* < 0.001). Diffuse and eggshell calcifications were less common in TB patients; however, the results were nonsignificant [7] and thus should be interpreted cautiously. One possible explanation for the pleural-based calcifications and thickening seen in *M. tuberculosis* is that fibrosis within the interobular connective tissue may extend to involve the pleura. Alternatively, tuberculous nodules may form within the pleural surface—similar to pleural plaques—followed by local connective tissue proliferation and calcification. These are mechanisms that may explain the extensive calcifications observed in tuberculosis cases.

## 5. Implications of Calcification in Granuloma Formation

**Figure 2:**
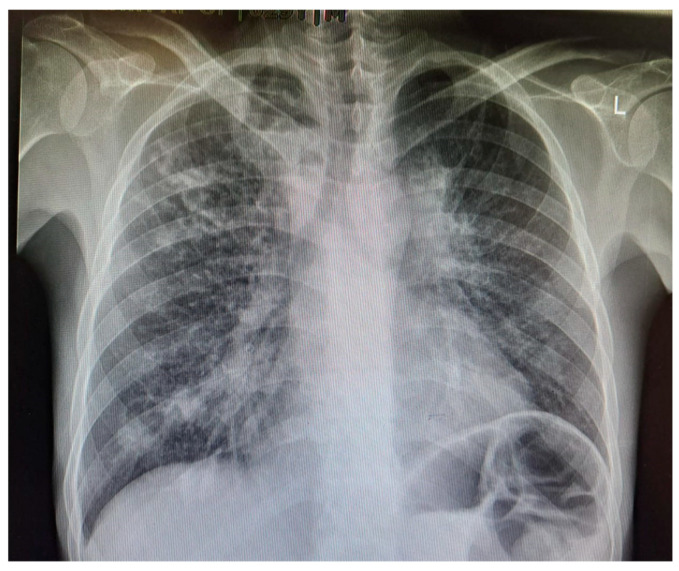
Chest X-ray of a 29-year-old male with active TB, showing bilateral upper lobe opacities and cavitation, consistent with advanced pulmonary TB. These findings reflect extensive lung parenchymal damage due to the infection.

**Figure 3:**
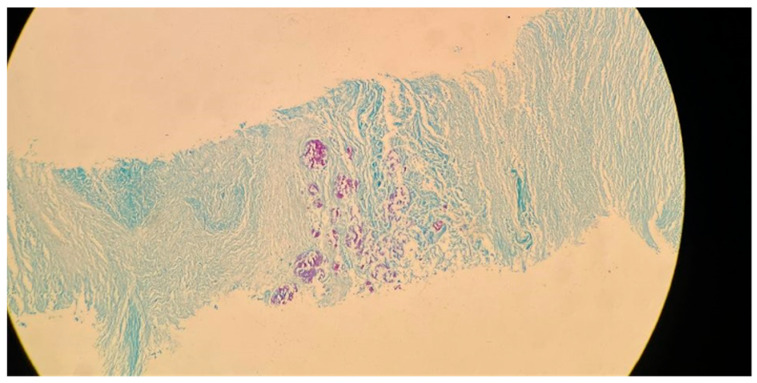
Acid-fast bacillus (AFB) staining of a sputum smear from the same patient referenced in Figure 2, demonstrating numerous acid-fast bacilli (red rods) against a blue background (100× magnification).

*M. tuberculosis* infection often leads to injury to the pleura. This includes the thickening and fibrosis of the pleura, and in many of these cases, patients further form calcification. About 40.9% of patients with fibrosis form these calcifications, as demonstrated in a CT-based study by Deshpande et al. [9]. The presence of fibrosis often results in decreased lung capacity on one side of the chest. This can be due to the cavities that form within the lungs as a result of the infection. In patients who had a single cavity in their lungs, fibrosis was found 100% of the time and calcification 80% of the time. In addition, 90% of these single cavities were found in the upper lobes of the lungs [9]. Upper lobe damage to the lungs can be seen in Figure 2. Further evidence of the presence of *M. tuberculosis* can be observed in Figure 3, in which many acid-fast bacilli are shown. The consistent presence of fibrosis and calcification in these cases suggests prolonged infection. This represents the host’s attempt to contain the mycobacterial focus. Fibrosis may contribute to disrupted lung structure and functional impairment of the lung, while calcification often marks the healing or inactive phase of a granulomatous lesion.

A total of 83–85% of *M. tuberculosis* infections tend to infect the apical or posterior areas of the upper lobes, likely due to the high levels of oxygen compared to the lower lobes. The higher concentration of oxygen allows for greater multiplication of the bacterium. Fibrosis of the lung tissue begins once the healing process starts. The resulting fibrosis and granulomas both impede normal airflow in the lungs. Difficulty in breathing is further exacerbated by the buildup of large levels of mucus. As the body tries to heal the tissue, calcium forms as a sequelae of chronic inflammation. Calcification from lymph nodes continues to increase, forming calcification of the bronchial tree, causing coughing and wheezing to worsen [10]. Although this process of broncholithiasis is uncommon, it highlights the potential downstream effects of chronic calcification.

One suggested molecular mechanism of fibrosis in *M. tuberculosis* infections is through macrophage-to-myofibroblast transformation. The suggestion is that macrophage-to-myofibroblast transformation is the basis for fibrosis caused by granulomas through M1 macrophages via both anti-inflammatory and inflammatory signaling in these granuloma macrophages. Evans et al. used systems biology to predict that macrophage-to-myofibroblast transformation could account for fibrosis observed in granulomatous lesions, particularly in areas of sustained immune activation [11]. Supporting this, Warsinske et al. identified key signaling pathways, including TGF-β, that may drive this transition and promote extracellular matrix deposition [12]. Although this mechanism is still under study, this provides evidence in support of macrophage-to-myofibroblast transformation being a key contributor to the progression from granulomatous inflammation to fibrotic lung disease in TB.

*M. tuberculosis* infections can have a variety of effects on disease progression. The calcification that forms is a result of both the host and the bacterium responding to each other. The host attempts to “wall off” the infection, while *M. tuberculosis* attempts to further multiply in this scenario [13,14]. This forms an immune equilibrium in which the host attempts to wall off the pathogen through granuloma formation, while the bacteria in turn are adapting to survive within the nutrient-limited environment. Fibrosis and calcification in TB reflect progressive stages of the host’s immune response. Initially, granulomas form to contain the bacilli. Over time, chronic inflammation drives fibrotic remodeling and, eventually, calcification—often signifying immune control. However, M. tuberculosis can persist within these lesions, and fibrosis may paradoxically reduce immune cell access, enabling bacterial survival for latency. In rare cases, a buildup of calcification can be followed by consequences such as tracheobronchial narrowing, pulmonary vessel encasement, superior vena cava obstruction, and pulmonary infiltrates [9]. It has been shown that early in the infection, lesions can become necrotic and calcify, and within two weeks, *M. tuberculosis* can be observed in lymph nodes near the lungs. The infection can further spread and reach other organs such as the spleen [14,15].

Long-term consequences may arise from infection with *M. tuberculosis,* even with treatment. An example of this can be seen with children, for whom there are no supported drug cocktails for long-term use since their infections typically display resistance after transmission from adults [16,17]. Tuberculosis is also recognized as a long-term risk factor for the development of chronic respiratory diseases, such as COPD and post-tuberculosis lung disease. Several cohort studies have shown that TB survivors exhibit persistent airflow obstruction, even years after microbiologic cure, which is often misdiagnosed as COPD [18,19,20,21,22,23,24,25,26]. This underscores the long-term burden of fibrosis, as it predisposes individuals to reduced lung function, recurrent infections, and increased mortality. For those who have had treatment against *M. tuberculosis*, the presence of lymphadenopathy can either dissipate, or sometimes a residual mass can remain made up of the fibrosis and calcification in the tissue. This remaining calcification can appear in various ways including pleural calcification, pleural thickening, and fibrothorax. These calcified scars, often referred to as “healed granulomas”, can represent the resolution of active disease. However, similar to keloids or post-burn scarring, the fibrotic changes accompanying calcification may impair pulmonary function. If too much time is spent waiting before treatment is initiated, greater spread and advancement of *M. tuberculosis* can occur, potentially leading to more of these lesions remaining post treatment [27]. The existence of fibrosis because of inflammation and the healing process may increase the probability of future lung complications. The body’s ability to have a balance of inflammation and anti-inflammatory effects may be upset, resulting in further damage via the scarring of tissue/fibrosis. The specific use of rifampicin and isoniazid may also lead to fibrosis, as well as drug-induced pneumonitis [19,20]. Although rare, the drugs’ role in promoting fibrosis remains controversial and should not detract from their essential role in a tuberculosis treatment regimen.

## 6. Cytokine Signaling and Fibrotic Remodeling in Tuberculosis

Multiple cytokines have been implicated in the fibrotic response observed in *M. tuberculosis* infection. TNF-α has been shown to influence collagen metabolism, with increased expression correlating with increased fibrosis. TGF-β signaling pathways have been observed both before and after treatment in patients with pulmonary TB, suggesting a sustained role in tissue remodeling and repair. Elevated IL-1β levels have also been associated with larger cavity formation, potentially causing fibrosis [4].

In addition to these cytokines, IL-4, IL-33, and TGF-β produced by activated dendritic cells have been linked to airway fibrosis [18]. TGF-β1 plays a critical role by inducing fibroblast differentiation into myofibroblasts—key effectors in extracellular matrix deposition. This process is counterbalanced by IL-10, which inhibits myofibroblast activation and collagen production. While the presence of TGF-β1 near fibroblasts is required for fibrogenesis, it is insufficient on its own, suggesting that additional signaling inputs are necessary for the full development of fibrosis [12].

## 7. Pulmonary Function and Calcification-Induced Fibrosis

Damage to the lung parenchyma from *M. tuberculosis* infection is not solely due to granulomas. In TB, cytokines such as TGF-β and IL-10 play complex roles: TGF-β promotes fibroblast-to-myofibroblast differentiation, contributing to ECM deposition, while IL-10 modulates inflammation. Elevated TGF-β has been observed in both active and post-treatment *TB* lungs [28]. Given that the lung epithelium is a delicate single-layer structure, it is easy to recognize that excess deposition of collagen can easily interfere with normal physiological processes. Excess collagen deposition, or parenchymal fibrosis, occurs during the healing or chronic phase of tuberculosis [28]. Zong and Ouyang described a case report of mediastinal fibrosis and pulmonary hypertension secondary to *M. tuberculosis* infection [29], which was due to lymph node involvement and distinct from parenchymal fibrosis. While such cases are uncommon and largely limited to individual case reports, they underscore the potential for tuberculosis to cause long-term structural and vascular complications that impair respiratory function, even after microbiological resolution of the disease. This demonstrates the possible long-term effects of *M. tuberculosis* infection that can alter respiratory function even well after the resolution of any active infection.

Matrix metalloproteinases (MMPs) have also been largely implicated as key players in *M. tuberculosis* infection and the formation of granulomas and cavitations [4]. MMPs encompass a family of zinc-dependent endopeptidases that can catalytically cleave varying substrates such as collagen, gelatin, and basement membrane proteins [30]. In one study involving 108 *M. tuberculosis*-infected patients, neutrophils were shown to directly secrete MMP-8, and MMP-8 levels were shown to be closely associated with the severity of *M. tuberculosis* infection as it relates to both clinical and radiographic findings [31]. Of note, the study also found that within the sputum of *M. tuberculosis*-infected patients, they had approximately a 5-fold increase in the levels of MMP-8 and collagenase activity, lending to the degradation of healthy lung tissue, thus contributing to the damage of the lung architecture. MMP-2, MMP-3, MMP-7, and MMP-9 have all been deemed as profibrotic [32].

**Figure 4:**
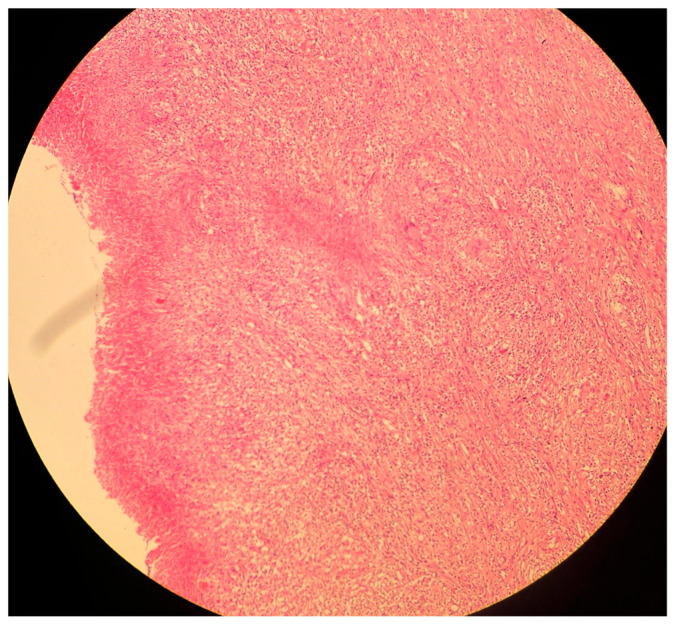
A histological image of a necrotizing granuloma from a pleural biopsy sample of a patient with TB (51 years old /male). The sample is stained with hematoxylin and eosin (H&E), highlighting the central area of caseous necrosis surrounded by epithelioid macrophages, multinucleated giant cells, and a rim of lymphocytes (10× magnification). This structure is characteristic of granulomatous inflammation seen in TB.

The short and long-term sequelae of *M. tuberculosis* infection often relate to their specific effects on the lung architecture (Figure 4). Granulomas are space-occupying lesions which take up volume within lung parenchyma that normally require as much surface area as possible to function properly (Figure 4). Fibrosis leads to decreased elasticity in the lungs, causing a hindrance of lung recoil that serves an important role, primarily during exhalation. Matrix metalloproteinases serve an important function throughout the body, but can also cause severe damage to the lungs, especially during *M. tuberculosis* infection.

### 7.1. Long-Term Pulmonary Dysfunction Following TB-Induced Remodeling

**Figure 5:**
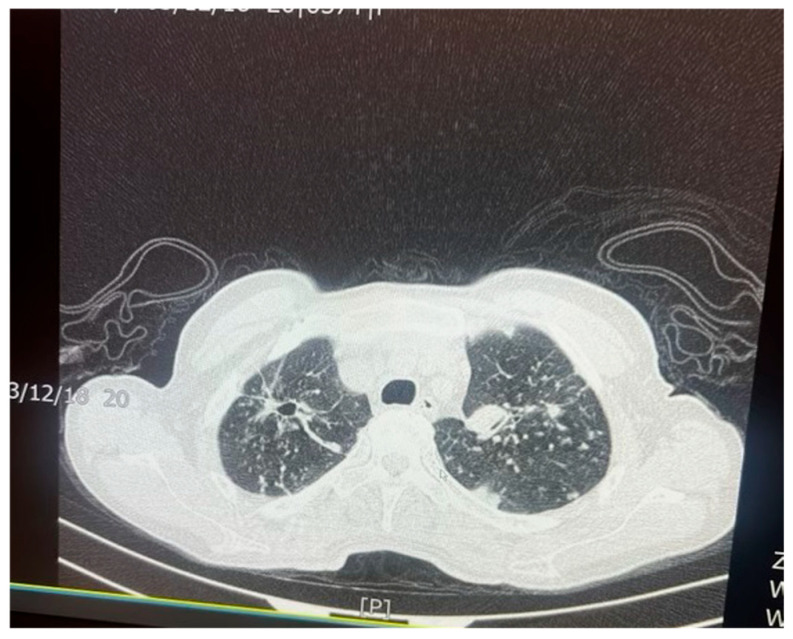
An axial chest CT scan of a 57-year-old female patient with pulmonary TB, showing bilateral pulmonary cavities and multiple nodules. The cavities are seen predominantly in the upper lobes, with irregular thickened walls, suggestive of active TB. The nodular lesions surrounding the cavities represent granulomatous inflammation, which is characteristic of TB infection. These findings are typical of post-primary TB in adults.

**Figure 6:**
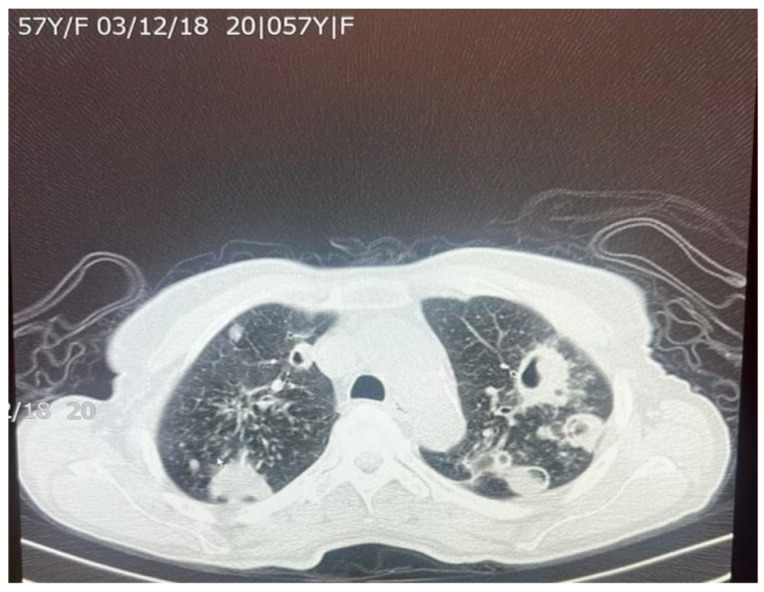
An axial chest CT scan of the same patient in Figure 5, demonstrating advanced nodular and cavitary changes in both lungs, consistent with pulmonary TB. The bilateral upper lobe cavities are clearly visible with associated nodules. The distribution of the lesions, with upper lobe involvement and cavitation, is indicative of reactivation of TB. Additionally, areas of tree-in-bud appearance suggest bronchogenic spread of the infection, which is common in TB patients.

*M. tuberculosis* can alter the normal lung architecture in cases of both latent and active disease and even cause permanent damage after resolution of the infection. Damage to lung tissue, such as the replacement of tissue with scar tissue, can permanently alter respiratory function, leading to significant morbidity well after the bacterium has been cleared. Figure 5 and Figure 6 illustrate the pattern of damage that points to the reactivation of TB. The cyclical process of tissue injury, immune-mediated inflammation, and healing contributes to fibrosis and calcification, which may lead to both obstructive and restrictive patterns of lung disease. Obstructive dysfunction can result from airway inflammation, loss of elastic recoil, and post-infectious bronchiectasis—the latter characterized by permanent dilation of the bronchi. On the other hand, restrictive changes arise from exaggerated immune responses and chronic inflammation, leading to fibrosis and stiffening of the lung parenchyma. The degree of respiratory mechanic abnormalities (i.e., pulmonary function tests) from *M. tuberculosis* infection can vary depending on multiple factors such as patient age, geographic location, or the drug resistance of the bacteria [25].

Lung parenchyma is normally elastic and possesses the ability to expand and recoil with each breath. Respiratory mechanics refer to several measures relating to pressure and flow that influence one’s overall lung function [33]. A few factors that are directly implicated in *M. tuberculosis* infection are lung compliance, volume, and work of breathing as they relate to fibrotic changes in the lung from chronic infection. One systematic review revealed a positive association between history of *M. tuberculosis* infection and the development of chronic airway obstruction that was independent of cigarette smoking and other confounding variables [26]. Another study found that 41% of participants, on average 31 years old, who had received drug-resistant *M. tuberculosis* treatment demonstrated significant lung impairment (FEV1/FVC ratio of <0.7) consistent with COPD, despite their young age. These findings were shocking when compared to a population survey that showed a prevalence of only 7–12% in a group that included people who were approximately 10 years older [23,34].

**Figure 7:**
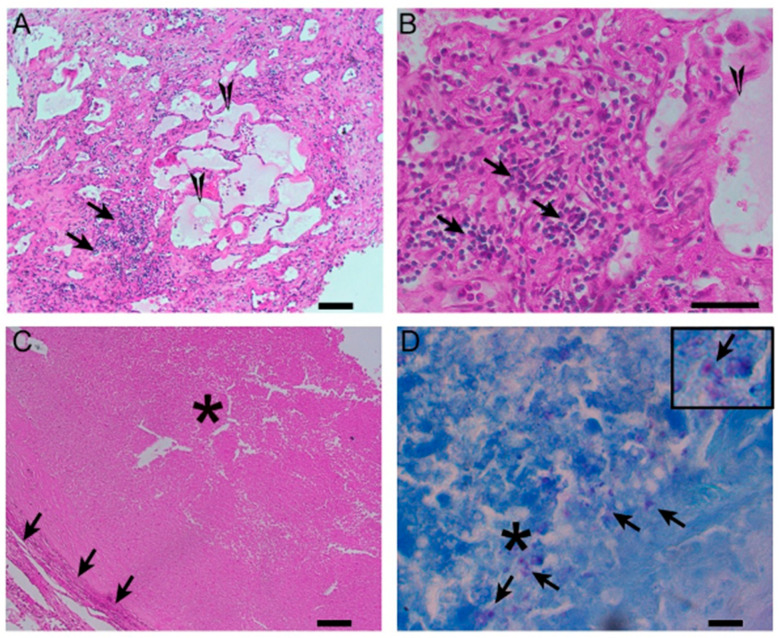
Different types of granulomas, including those comprising diffused immune cell aggregates and mild necrosis (**A**,**B**) or fibro-necrotic and caseating nodules (**C**), were noted on the TB lungs (40× magnification). The former type of granulomas were interspersed with pneumatic fluid accumulation in the lung parenchyma, which was surrounded by mononuclear and polymorphonuclear cells, resembling, respectively, the lymphocytes and neutrophils (**A**,**B**). In fibrotic nodules (**C**), the cellular debris was encapsulated by a fibrotic layer, composed of epithelioid histiocytes. At the core of these lesions, numerous *M. tuberculosis*-loaded cellular debris were noted ((**D**) and inset). These observations were consistent with and supported by our previous reports on TB lung granulomas in patients [37,38]. Arrows in panel (**A**,**B**) indicate diffused immune cell aggregates. Triangles in panel (**A**,**B**) denote mild necrosis. Arrows in panel (**C**) indicate fibrotic regions. Asteriks in panel (**C**) illustrate that cellular debris was encapsulated by a fibrotic layer, composed of epithelioid histiocytes. In panel (**D**), asteriks tllustrate the core of the debris lesions, and arrows denote lesions containing numerous *M. tuberculosis*-loaded cellular debris.

Granuloma formation tends to predominate during the post-primary TB stages and involves caseous necrosis and the development of cavitary lesions which are capable of eroding the airways, not only leading to impacts on respiratory mechanics but also increasing the spread of infection [35,36]. Figure 7 displays various kinds of granulomas that can form as a result of infection. The sequelae of infection eventually lead to worse gas exchange. Damage to specific regions of the lungs, whether it be upper, middle, or lower lobes, can affect essential respiratory physiology such as ventilation/perfusion ratios which can help determine states of hypoxemia.

Lung tissue collected via pneumonectomy among TB cases was fixed in neutral formalin followed by paraffin embedding (FFPE). These FFPE blocks were cut into 5-micron-thick slices, placed on charged glass slides (VWR) and used for staining with hematoxylin and eosin (H&E) or the Ziehl–Neelsen acid-fast staining (AFB) procedures to visualize host cells and bacteria, respectively, as described earlier [39]. The lung histopathology was analyzed microscopically using a Nikon Microphot-FX microscope with NIS elements software F3 version (Nikon Instruments Inc., Melville, NY, USA).

Among granulomatous infections, such as TB and sarcoidosis, dystrophic calcification represents a key sequela of tissue damage, particularly in pulmonary TB. Calcification can occur via two distinct pathways, metastatic or dystrophic. In brief, metastatic calcifications arise from previously healthy tissue and involve excess deposition of calcium, whereas dystrophic calcifications occur in tissue that has been previously damaged [40]. *M. tuberculosis* produces dystrophic calcifications which occur only in damaged or necrotic tissues and do not cause hypercalcemia, as often seen in metastatic calcifications.

The mechanism of calcification is primarily driven by the ectopic production of 1α-hydroxylase specifically expressed within macrophages, which is responsible for converting vitamin D into its active form calcitriol [41]. It is interesting to note that the prevalence of hypervitaminosis D in *M. tuberculosis*-infected patients is variable and that fluctuations in serum vitamin D are even influenced by the very drugs used to treat *M. tuberculosis* [42]. While often incidental, TB-associated calcifications can occasionally lead to dyspnea, underscoring their potential clinical burden [43].

### 7.2. Long-Term Consequences for Pulmonary Function

After *M. tuberculosis* and long-term inflammation, the overall architecture of the lungs becomes permanently damaged. As discussed above, a reduction in the overall number of functional alveoli for gas exchange results in an increased risk of hypoxia and dyspnea. Another important sequela of chronic inflammation and extensive fibrosis is an increased risk of lung cancer. Numerous cellular processes are involved in scar formation/fibrosis and this chronic state of damage and repair. Persistent reactive oxygen species production during chronic TB inflammation can induce DNA damage, contributing to fibrosis and oncogenesis [44]. Additionally, inflammatory cytokines such as IL-1, IL-6, and TNF-a help facilitate the release of angiogenic compounds such as vascular endothelial growth factor (VEGF) and TGF-β that can contribute to cancer development and proliferation [44,45]. Moreover, fibrotic tissue contributes little to gas exchange and may promote further immune evasion by impairing drug and immune cell penetration [46].

## 8. Diagnostic Approaches for Assessing Calcification and Fibrosis

Being able to properly diagnose fibrosis and accurately determine the stage it is in is crucial for providing effective treatment and therapies. The stage of fibrosis can also be useful in determining the progression of certain extrapulmonary diseases such as chronic kidney disease and fatty liver disease [47]. For these reasons, there is significance in determining the most suitable diagnostic approach for fibrosis and calcification in organs.

The long-standing method of diagnosing fibrosis is via biopsy; however, with its level of invasiveness and risk of complications, efforts have been placed on establishing non-invasive diagnostic methods [47]. HRCT is a key non-invasive tool in identifying fibrosis in TB-affected lungs, though smoking and age may confound interpretation [48,49]. However, HRCT does have its disadvantages. HRCT scanning takes longer than other methods and provides a limited view of the lung in certain planes [48]. Alternatively, another method of CT, known as spiral CT, appears to compensate for the shortcomings of HRCT [48]. Spiral CT provides a better 3D assessment of the lung parenchyma and airway abnormalities, as well as providing accurate recordings of lung volume and air trapping in all lung regions. These advantages come with the cost of higher radiation doses [48,50].

With regards to calcification, computed tomography (CT), has been the standard for diagnosis. Given the choice of using contrast-enhanced or unenhanced CT, it is wiser to elect the unenhanced form as the presence of contrast has the ability to hide the appearance of calcification, leading to a possible missed diagnosis. Magnetic resonance imaging (MRI) has also been suggested as a method for detecting calcifications. However, calcium does not have a uniform appearance on most MRI scans, instead presenting with varying signal intensities. This makes MRI less reliable for detecting calcification and thus CTs remain the most sensitive method of detecting calcium [51].

Another non-invasive method of identifying and monitoring fibrosis is magnetic resonance imaging (MRI). A study conducted on infants and young children with cystic fibrosis (CF) aimed to identify patients with early structural lung damage who would qualify for early therapeutic intervention. The method of identification was MRI, which had previously proven to be a successful asset in identifying abnormalities in older patients without exposing them to unnecessary radiation. Researchers found that MRI was able to successfully identify lung structure and perfusion abnormalities, as well as monitor the changes experienced by the young patients throughout the course of their therapies, making it a suitable technique for monitoring fibrosis [52].

The use of MRI as a diagnostic tool has also been proven useful through its ability to detect inflammatory responses during the early stages of disease, along with identifying fibrosis in organs such as the liver, kidneys, heart, and lungs. In the kidneys, the more common types of MRI include diffusion-weighted imaging (DWI) and blood oxygen level-dependent (BOLD) MRI, which are protective against nephrogenic systemic fibrosis as they omit gadolinium-based contrast agents [47]. In the lungs, MRI is the most common method used to diagnose pulmonary fibrosis and can distinguish blood vessels in both fibrotic and healthy lungs through pulmonary angiography [47].

Overall, it appears that CT and MRIs are both effective and prominent diagnostic tools when identifying fibrosis and calcification in organs and tissues. Their non-invasive nature places them as an attractive alternative to the long-standing method of biopsy. Researchers continue to seek ways to optimize the use of these methods, and future direction indicates that the identification of more sensitive and specific biomarkers will strongly aid these present diagnostic tools [47].

## 9. Therapeutic Approaches and Intervention Strategies

Chronic fibrosis can occur in numerous organs, including the kidneys, liver, heart, and lungs. Up to 45% of deaths can be attributed to fibrosis in industrialized countries [53]. This process is marked by excessive ECM deposition and overactivation of myofibroblasts in response to dysregulated signaling pathways (FGF, PDGF, TGF-β, etc.). Key players that can be addressed with medications include inflammatory cells such as macrophages, inflammatory mediators (i.e., TNF-α), and how certain cells/compounds interact with the ECM [54]. TNF-α has been shown to serve an important role in cardiac fibrosis by promoting the differentiation of endothelial cells into mesenchymal cells, a process sometimes referred to as endothelial-to-mesenchymal transition (EMT) [55]. One might think that the administration of anti-TNF-α antibodies would counteract the fibrotic processes within the lungs and potentially improve fibrosis. Their utility has been well established for many autoimmune diseases, but these treatments (i.e., Adalimumab, Infliximab) are not efficacious for pulmonary etiologies and have even led to reactivation of *M. tuberculosis* infection and worsening of pulmonary outcomes [56,57]. This presents a significant dilemma in tuberculosis survivors, where the need to manage pulmonary fibrosis must be balanced against the risk of tuberculosis reactivation through immunosuppression.

Other additional treatments include mycophenolate mofetil (MMF), which works by decreasing the availability of guanosine nucleotides for B and T lymphocytes via blockade of inosine-5′-monophosphate dehydrogenase (IMPDH), and rituximab, a monoclonal anti-CD-20 antibody that prevents B lymphocyte differentiation into plasma cells and is used as second-line treatment [58,59]. Studies on patients with systemic sclerosis have revealed that after 2 years of MMF treatment, statistically significant improvements in dyspnea were observed, as well as in DLCO percentage of predicted outcome and FVC% of predicted outcome when compared to placebo [60].

These current treatments have been shown to be effective for other diseases, such as IPF, and further studies are required to see how well these may translate into other fibrotic processes. As with many immunomodulating therapies, there is an increased risk of reactivation of latent *M. tuberculosis* infection.

### 9.1. Novel Interventions Under Investigation

#### 9.1.1. Antifibrotic Agents

Myofibroblasts serve as an extremely important cell type when it comes to tissue healing and scar formation by means of deposition of the ECM and induction of tissue contraction. However, under chronic activation, this leads to fibrosis and organ dysfunction. Novel therapeutics are currently under investigation and hope to either target apoptotic processes or target some of the many modulators of myofibroblast differentiation, activation, etc.

Nintedanib and pirfenidone have become two FDA-approved medications for the treatment of idiopathic pulmonary fibrosis (IPF) [61]. Nintedanib is an intracellular tyrosine kinase inhibitor that helps prevent the functions of fibroblasts and several growth factors, including vascular endothelial growth factor (VEGF), fibroblast growth factor (FGF), and platelet-derived growth factor (PDGF) [62]. These treatments have demonstrated their ability to slow disease progression in patients with IPF by approximately 50% [63]. Pirfenidone is a small, synthetic molecule with a mechanism of action that is not entirely understood but is involved in interfering with the signaling pathways of TGF-β and other growth factors [64]. Although these therapies have been approved for the use of IPF, unfortunately, there have been several reported cases of *M. tuberculosis* reactivation while taking these medications [65,66]. This creates a challenging circumstance due to the fact that these therapies can combat fibrotic processes caused by *M. tuberculosis* infection yet have the capability of leading to worsening infection or reactivation of latent *M. tuberculosis*.

Another possible treatment that has demonstrated antifibrotic properties is derivatives of artemisinin, a plant-derived compound used primarily as a chemotherapeutic and anti-malarial drug. In rat models, artesunate (semi-synthetic derivative of artemisinin) has been shown to inhibit many profibrotic processes of multiple tissue types both in vitro and in vivo, including prevention of fibroblast differentiation, induction of myofibroblast apoptosis, and inhibition of ECM deposition [67]. Artemisinin and artesunate were compared against common first-line anti-tubercular drugs (rifampicin, isoniazid, ethambutol) in *M. tuberculosis*-infected rats through numerous assays and in vivo tests. Surprisingly, artesunate performed better than artemisinin, effectively inhibited *M. tuberculosis* growth in a concentration-dependent manner, and even continued to strongly inhibit the growth/proliferation of *M. tuberculosis* 21 days after initial treatment [68]. These promising results indicate the utility that artemisinin and similar compounds possess as either standalone therapy for *M. tuberculosis* or in synergy with first-line therapies.

#### 9.1.2. Immunomodulatory Approaches

Current approaches utilized to combat *M. tuberculosis* infection include the conventional chemotherapeutic drug regimen of isoniazid, rifampin, and pyrazinamide. A study performed on guinea pig models receiving this conventional treatment regimen for 19 weeks demonstrated a marked reduction in bacterial loads within one month of treatment. Even after this, residual calcified lesions were noted as well as extracellular bacilli, which could suggest regions of reactivation of the disease despite anti-tubercular treatment [69]. Conventional tuberculosis therapy reduces bacterial load, but that study shows that residual fibrotic lesions often persist, prompting investigation into immunomodulatory agents that can prevent long-term pulmonary damage. Other treatments under investigation include bone marrow-derived mononuclear cell (BMDMC) therapy. Hematopoietic and mesenchymal stem cells found within the bone marrow possess an abundance of progenitor cells which facilitate cellular repair and remodeling of damaged tissues.

In a case series of three patients with severe asthma who received a single dose of autologous BMDMCs, although no major improvements in overall lung function were noted, all patients demonstrated improvements in subjective quality of life surveys soon after treatment termination [70].

Although studies in murine models show that BMDMCs reduce fibrosis and inflammation, there is insufficient evidence for their efficacy in active TB with a large bacterial load. BMDMCs appear to function primarily through the modulation of several paracrine processes, yielding improvements in lung mechanics and reductions in several parameters, including the number of inflammatory cytokines, the number of macrophages present in alveolar septa, alveolar collapse, and collagen fiber content within the lung parenchyma. In one murine model of silicosis, the administration of BMDMCs reduced the fraction area of granuloma by 144% [71]. Another study found that mice subjected to acute lung injury (ALI), either by intratracheal or intraperitoneal methods, who received BMDMCs showed increased tissue repair, higher survival rates, and decreased cellular apoptosis [72]. Although there are not many human studies looking at BMDMC treatment for pulmonary pathologies, BMDMC therapy has been studied against other fibrotic diseases. In a meta-analysis looking at randomized–controlled trials involving patients who had non-ischemic dilated cardiomyopathy, treatment with BMDMCs resulted in a moderate yet statistically significant improvement in their left ventricular ejection fraction. Although that study focused on cardiac fibrosis, the molecular signaling and pathophysiology are very similar across the different tissue types. This treatment regimen is promising but requires trials in TB-specific models before therapeutic translation.

Administration of IFN-γ either as monotherapy or given alongside other treatments has also demonstrated promising results. As described earlier, IFN-γ is an important cytokine that is integral to numerous immunomodulatory processes, including antifibrotic capabilities. The effective eradication of *M. tuberculosis* involves a Th-1 immune response, which involves intracellular pathogens. IFN-γ is a key cytokine within the Th-1 pathway and therefore has become a potential focus for better understanding effective treatments for *M. tuberculosis* infection. IFN-γ has been shown to act synergistically with pirfenidone to successfully tackle lung fibrosis by preventing fibroblast proliferation and differentiation [73]. In a meta-analysis of nine clinical trials looking at using IFN-γ as adjuvant therapy, it was found that aerosolized administration (three times a week) resulted in statistically significant improvements in sputum negative conversion and improvements in chest radiographs [74]. An older study including five patients with multidrug-resistant *M. tuberculosis* found that after 1 month of IFN-γ treatment, there was complete resolution of infection according to acid-fast bacillus sputum smears, and pulmonary cavitary lesion size decreased across all patients [75]. Of note, while aerosolized administration has been deemed effective, subcutaneous delivery has not been shown to be efficacious [76].

## 10. Challenges and Future Directions

One of the major challenges in addressing calcification and fibrosis resulting from *M. tuberculosis* infection lies in the complexity of the underlying pathophysiological mechanisms and the resulting diversity of clinical presentations. Calcification and fibrosis are multifactorial processes involving numerous cellular, molecular, and immunological pathways, and each patient may present with variations based on age, immune status, and comorbidities [4,11]. The interplay between host responses, such as the immune system’s attempt to contain the infection through granuloma formation, and bacterial mechanisms for adapting and thriving in hostile environments is not yet fully understood. For instance, macrophage-to-myofibroblast transformation is an area of significance, as the exact stimuli that lead macrophages to adopt profibrotic roles are still under investigation [11]. Future research will need to employ advanced technologies, such as single-cell RNA sequencing and high-dimensional flow cytometry, to dissect these cellular behaviors and elucidate the precise signaling cascades involved.

Another challenge involves the limitations of current diagnostics. While imaging techniques like high-resolution computed tomography (CT) and magnetic resonance imaging (MRI) are helpful in detecting and monitoring fibrosis and calcification, they come with significant limitations. For instance, high-resolution CT is valuable for its clarity in imaging lung structures but involves radiation exposure, which is a concern for repeated use, especially in younger populations or those with comorbidities [9]. MRI, on the other hand, is less reliable for detecting calcification due to variable signal intensities on T1- and T2-weighted sequences [10]. Future research could involve the development of molecular MRI techniques that target specific biomarkers of calcification or fibrosis within lung tissues, potentially allowing for more accurate non-invasive monitoring of disease progression.

Therapeutically, the treatment of calcification-induced fibrosis remains hindered due to the risk of reactivating latent TB. Antifibrotic drugs, such as nintedanib and pirfenidone, which are used in idiopathic pulmonary fibrosis, show potential but are associated with risks of *M. tuberculosis* reactivation [12]. There is a need for new therapies that suppress fibrosis without impairing immune control of infection. Plant-derived compounds with antifibrotic effects, demonstrated in preclinical studies, may offer such promise.

One promising avenue for future therapeutic research lies in immunomodulation, such as the use of cytokine therapies with interferon-gamma (IFN-γ). IFN-γ, a key Th1 cytokine, may exert antifibrotic effects and act synergistically with existing antifibrotic agents [73]. However, these approaches are still largely experimental, and their application in *M. tuberculosis*-infected populations needs rigorous validation. Future research should include randomized controlled trials to establish optimal dosing, delivery methods, and long-term outcomes for these interventions.

In conclusion, addressing post-tuberculosis calcification and fibrosis requires a deeper understanding of the underlying mechanisms, better diagnostics, and safer therapeutics. Future efforts should combine emerging technologies with novel treatment strategies to improve long-term outcomes for affected individuals.

## 11. Discussion

### 11.1. Granuloma Dynamics and Impacts

Granuloma formation plays a crucial role in the pathogenesis of *M. tuberculosis*. It is a dynamic process that begins when alveolar macrophages phagocytose *M. tuberculosis*. These macrophages release various cytokines such as TNF-α and IL-12 that recruit additional immune cells such as neutrophils, T cells, B cells, and dendritic cells to the site of infection. The accumulation of these cells is what leads to a granuloma, a structure designed to sequester the infections away from healthy cells and prevent its spread. Granulomas take up space and interfere with the function of normal lung parenchyma [77]. Although sequestering infection away from healthy tissue sounds like a positive, granulomas can have lasting permanent negative effects as well. Granulomas can lead to bacteria developing a persistent dormant state. This is hard to control in individuals with latent TB, as the disease cannot spread but can be reactivated at a later time. Granulomas can lead to liquefactive necrosis, which leads to an air-filled cavity that prevents gas exchange [4].

### 11.2. Calcification: Mechanisms and Implications

Calcification is another sequela of chronic *M. tuberculosis* infection. It can occur via metastatic or dystrophic pathways. Dystrophic calcification occurs in previously damaged tissues without systemic hypercalcemia. The mechanism of calcification is primarily driven by the ectopic production of 1α-hydroxylase specifically expressed within macrophages which is responsible for converting vitamin D into its active form calcitriol [41]. While calcified granulomas are often considered markers of controlled or resolved infection, they can be clinically significant. Large calcifications may contribute to tracheobronchial narrowing or superior vena cava obstruction, exacerbating respiratory symptoms such as dyspnea and coughing [10].

### 11.3. Challenges in Diagnosis and Management and Future Directions

Accurate diagnosis of granuloma-associated calcification and fibrosis remains difficult. CT and MRI are commonly employed to detect abnormalities. However, the limitations of these modalities do pose a problem—radiation exposure in CT and MRI being unable to detect calcification due to varying signal intensities [51].

Antifibrotic agents like nintedanib and pirfenidone have shown efficacy in idiopathic pulmonary fibrosis [61,62], but their utility in *M. tuberculosis*-related fibrosis is diminished by latent *M. tuberculosis* reactivation. Immunomodulatory therapies, including interferon-γ (IFN-γ) and bone marrow-derived mononuclear cells (BMDMCs), offer potential alternatives by targeting specific pathways involved in fibrosis and the immune system. However, these interventions require further evaluation through clinical trials to establish their efficacy. This could perhaps be explored in a follow-up study.

In conclusion, the pathogenesis of calcification and fibrosis in *M. tuberculosis* infection is a complex process with significant implications for patient outcomes. Advancing our understanding of these processes through research is essential to improve the quality of life for individuals affected by TB.

## 12. Conclusions

*M. tuberculosis* infection can lead to long-term pulmonary sequelae. Fibrosis and calcification can significantly contribute to morbidity even after infection is cleared clinically. Granulomas, while initially protective, often result in chronic inflammation and tissue remodeling, which underly these pathological changes. Current treatments, including corticosteroids and antifibrotic agents such as nintedanib and pirfenidone, show promise in slowing fibrotic progression, but their application in TB-related fibrosis remains limited by the risk of latent *M. tuberculosis* reactivation.

Novel approaches, such as artemisinin derivatives, IFN-γ therapy, and bone marrow-derived mononuclear cells (BMDMCs), offer exciting avenues for dual-action therapies that both suppress fibrosis and bolster immune defense. Artemisin derivatives are being evaluated in preclinical models for their dual anti-fibrotic and antimycobacterial effects. BMDMCs have demonstrated beneficial outcomes in animal models of tuberculosis-induced lung fibrosis. IFN-γ therapy has been tested in small clinical studies for multidrug-resistant TB, showing potential to reduce disease burden.

Despite progress in understanding the mechanism behind post-*M. tuberculosis* fibrosis and calcification, significant gaps remain. A deeper grasp of immune signaling, cellular transition, and the tissue remodeling processes involved—in the context of host immune response variability—will be critical. Future research must focus on developing targeted therapies that not only limit fibrotic damage but also preserve or enhance host immunity to prevent *M. tuberculosis* latent reactivation. By addressing these challenges, the field moves closer to improving the prognosis and quality of life for individuals recovering from tuberculosis infections.

## Data Availability

No new data were generated or analyzed in this study. All patient images included are presented for illustrative purposes with proper consent and ethical approval. The remaining data discussed in this review are derived from previously published studies, which are cited accordingly.
